# The initial assessment of expert panel performance in core hospitals for cancer genomic medicine in Japan

**DOI:** 10.1007/s10147-020-01844-1

**Published:** 2021-01-01

**Authors:** Kuniko Sunami, Yoichi Naito, Eriko Aimono, Toraji Amano, Daisuke Ennishi, Hidenori Kage, Masashi Kanai, Keigo Komine, Takafumi Koyama, Takahiro Maeda, Sachi Morita, Daisuke Sakai, Shinji Kohsaka, Katsuya Tsuchihara, Takayuki Yoshino

**Affiliations:** 1 Department of Laboratory Medicine, National Cancer Center Hospital, Tokyo, Japan; 2 Department of Breast and Medical Oncology, National Cancer Center Hospital East, Kashiwa, Japan; 3 Genomics Unit, Keio University School of Medicine, Tokyo, Japan; 4 Clinical Research and Medical Innovation Center, Hokkaido University Hospital, Sapporo, Japan; 5 Department of Hematology and Oncology, Okayama University Hospital, Okayama, Japan; 6 Department of Respiratory Medicine, The University of Tokyo Hospital, Tokyo, Japan; 7 Department of Therpeutic Oncology, Graduate School of Medicine, Kyoto University, Kyoto, Japan; 8 Department of Clinical Oncology, Tohoku University Hospital, Sendai, Japan; 9 Department of Experimental Therapeutics, National Cancer Center Hospital, Tokyo, Japan; 10 Department of Hematology, Oncology and Cardiovascular Medicine, Kyushu University Hospital, Fukuoka, Japan; 11 Department of Clinical Oncology and Chemotherapy, Nagoya University Hospital, Nagoya, Japan; 12 Center for Cancer Genomics and Personalized Medicine, Osaka University, Suita, Japan; 13 Section of Knowledge Integration, Center for Cancer Genomics and Advanced Therapeutics, National Cancer Center, Tokyo, Japan; 14 Division of Translational Informatics, National Cancer Center Exploratory Oncology Research and Clinical Trial Center, Tokyo, Japan; 15 Department of Gastrointestinal Oncology, National Cancer Center Hospital East, 6-5-1, Kashiwanoha, Kashiwa-shi, Chiba, 277-8577 Japan

**Keywords:** Precision oncology, Comprehensive genomic profiling tests, Expert panel, Genetic counseling, Investigational new drug trials, Core hospitals

## Abstract

**Background:**

Since June 2019, cancer genomic profiling (CGP) tests have been reimbursed by the National Health Insurance system in Japan, with restrictions for government-designated hospitals with a molecular tumor board composed of multidisciplinary specialists, known as an expert panel (EP). The standardization of EPs is a critical challenge for implementing precision oncology in the clinical setting.

**Methods:**

Data on consecutive cases who underwent the CGP tests at 11 core hospitals between June 2019 and January 2020 were collected. We evaluated the proportions of cases that received genomically matched treatments, including investigational new drugs (INDs) based on CGP results, and/or for which genetic counseling was recommended. Two simulated cases were annotated by each EP. The annotated reports were then centrally assessed.

**Results:**

Each EP mainly discussed the applicability to genomically matched treatments and the necessity of performing genetic counseling. A pre-review of the report by key members in each EP reportedly made the EP conference more interactive and efficient, and thereby saved time. A total of 747 cases underwent CGP tests, 28 cases (3.7%) received genomically matched treatment, and 17 cases (2.3%) were referred for genetic counseling. Annotated reports for the simulated cases varied across the EPs, particularly the number of recommended IND trials, which seemed to be associated with the actual number of participants in IND trials.

**Conclusions:**

This investigation provides reference data for the application of precision oncology in a clinical setting. Further investigations on the standardization of clinical annotations are warranted.

**Supplementary Information:**

The online version contains supplementary material available at 10.1007/s10147-020-01844-1.

## Introduction

Marked advances in precision oncology over the past two decades have made genotyping mandatory for most advanced cancer patients, as it helps to ensure proper therapy selection. Indeed, the National Comprehensive Cancer Network (NCCN) and European and Japanese Societies of Medical Oncology (ESMO and JSMO) practice guidelines now include recommendations for genotyping to guide therapy selection in at least 16 different types of cancer [1–3]. Despite this impressive progress, the pace of precision oncology innovations remains limited due to the daunting logistical realities of patient identification: many actionable targets are present in only a small fraction of patients, which means that hundreds or even thousands of patients need to be screened.

With the aim of implementing precision oncology in Japan, reimbursement has been provided by the National Health Insurance System for two comprehensive genomic profiling (CGP) tests, the OncoGuide NCC Oncopanel System [4] and the FoundationOne CDx Cancer Genomic Profile [5], since June 2019 [6]. The indication is restricted to patients with advanced solid tumors with disease progression during standard therapies or for whom there are no appropriate standard treatments, including rare cancers and carcinoma of unknown primary [6, 7]. In addition, for the purpose of consolidating precision oncology with quality control/assurance in designated hospitals, the Ministry of Health, Labour and Welfare (MHLW) originally designated 11 cancer genomic medicine core hospitals and 100 cooperative hospitals in April 2018 [8]. Reimbursement for CGP tests is restricted to these designated hospitals [6, 9].

Furthermore, before CGP test results are provided to the patient by an attending physician, these must be annotated by a regularly held intra-institutional molecular tumor board, called an expert panel (EP), which consist of multidisciplinary specialists at each core hospital, including medical oncologists, pathologists, genome researchers, medical geneticists, and genetic counselors, which are mandatory for a hospital to be designated as a core hospital. On the other hand, an attending physician working in a cooperative hospital should participate in regular EP meetings at the core hospital to which it is tied [6]. The EP meeting evaluates the pathogenicity of detected variants, the recommendations of genomically matched treatments, including investigational new drugs (INDs) and/or the necessity of genetic counseling based on the results of CGP tests [6, 10]. Because clinical annotations made by the EPs may potentially affect the patient’s actual treatment, the standardization of the EPs with quality control/assurance would be needed for implementing precision oncology in Japan.

In addition, the National Cancer Center (NCC) established the Center for Cancer Genomics and Advanced Therapeutics (C-CAT) in June 2018, which aggregates both the genomic data and clinical information of all patients who receive CGP tests in the clinical practice setting, to establish a central repository system [11]. The C-CAT also provides all of the core and cooperative hospitals with a clinical annotation report, called “C-CAT Findings” for each patient, which potentially make it easier to identify genomically matched treatments and to support the standardization of clinical annotation across EPs. However, the differences in clinical annotation among EPs have not been evaluated. We, therefore, conducted a survey of EPs at each core hospital and assessed the performance of EPs using two simulated cases.

## Methods

### Composition of membership and survey to investigate the operation and performance of EPs

The investigators, who were representatives of each EP at all of the core hospitals, are nominated. On February 27, 2020, an investigator meeting was held and the operation and performance of EPs was discussed. As a pre-meeting survey, we collected data on consecutive cases in which CGP tests were performed at all core hospitals between June 2019 and January 2020 and evaluated the number of cases that received genomically matched treatments and in which patients were referred for genetic counseling according to the recommendation by the EP. After consultation with the NCC Institutional Review Board (IRB), the IRB officially confirmed that the current study does not require IRB approval.

### Simulated case preparation

Two simulated cases were developed by medical oncologists (KS, YN and TY) at the National Cancer Center Hospital (NCCH) and National Cancer Center Hospital East (NCCHE), which are the most experienced core hospitals and which have performed the largest number of CGP tests thus far.

Case 1 was a male with advanced colorectal cancer who had failed to respond to standard chemotherapies. The patient harbored the following somatic genetic alterations as follows: *BRAF* V600E, *ATM* R35*, *NF1* Y1521*, *TP53* R273H, *APC* c.1312+1G>A, *ARAF* R326* and *NTRK2* L138P, and *BRCA2* V208G germline variant. Case 2 was a female with advanced breast cancer whose disease progressed on standard therapies, who showed *PIK3CA* H1047R, *ERBB2* S310Y and *CCND1* amplification. Cases 1 and 2 were tested by the OncoGuide NCC Oncopanel System and FoundationOne CDx Cancer Genomic Profile, respectively (Table 1). Simulated CGP result reports of these two cases were prepared by the medical oncologists (KS, YN, and TY), and simulated C-CAT Findings were prepared by C-CAT. Then, these reports (clinical history, results of the simulated CGP test and simulated C-CAT findings) were delivered to all EPs at core hospitals on December 24, 2019.

**Table 1:** Clinical information and the CGP results of simulated cases

	Case 1	Case 2
Gender	Male	Female
Age	50s	40s
Tumor type	Colon cancer	Breast cancer (ER+, PgR+, HER2 1+)
Previous chemotherapies		
1st line	FOLFOX + bevacizumab	Anastrozol
2nd line	FOLFIRI + cetuximab	Fulvestrant + palbociclib
3rd line	Investigational drug	Paclitaxel + bevacizumab
4th line	Regorafeinib	Eriblin
5th line	–	Capecitabin
6th line	–	Doxorubicin + cyclophosphamide
Family history of cancer	Mother: breast cancer (40s)	N/A
	Sister: breast cancer (50s)	
Medical history	Hypertension	N/A
Type of GCP test	OncoGuide™ NCC oncopanel	FoundationOne^®^ CDx
Detected SNVs and CNVs	Somatic variants	
	*BRAF* V600E	*PIK3CA* H1047R
	*ATM* R35*	*ERBB2* S310Y
	*NF1* Y1521*	*CCND1* amplification
	*TP53* R273H	
	*APC* c.1312+1G>A	
	*ARAF* R326*	
	*NTRK2* L138P	
	Germline variant	
	*BRCA2* V208G	

*N/A* not applicable, *CGP* comprehensive genomic profiling, *SNV* single-nucleotide variant, *CNV* copy number variant, *FOLFOX* oxaliplatin, folinic acid and 5-fluorouracil, *FOLFIRI* irinotecan, folinic acid and 5-fluorouracil, *BRAF* V-Raf murine sarcoma viral oncogene homolog B1, *ATM* ataxia telangiectasia mutated, *NF1* neurofibromatosis type I, *TP53* tumor protein p53, *APC* adenomatous polyposis coli, *ARAF* V-Raf murine sarcoma 3611 viral oncogene homolog 1, *NTRK2* neurotrophic tyrosine receptor kinase 2, *BRCA2* breast cancer susceptibility gene 2, *PIK3CA* phosphoinositide-3-kinase, catalytic, alpha polypeptide, *ERBB2* human epidermal growth factor receptor 2, *CCND1* cyclin D1

### Annotation by EPs for simulated cases

Each EP discussed the simulated CGP test results and simulated C-CAT findings and made recommended genomically matched treatment according to the evidence level and necessity of genetic counseling during their routine EP meetings, between December 25, 2019 and January 31, 2020. Note that the developers of simulated cases (KS, YN, and TY) never joined in the discussion at the EP meetings for these cases. The evidence level was defined in “Consensus clinical practice guidance for the CGP tests,” which was issued by three cancer-related major Japanese societies in October 2017 [12]. The annotated reports from each EP were centrally assessed by the developers (KS, YN, and TY) before the investigator meeting held on February 27, 2020.

## Results

### The operation of EPs at each core hospital

At the investigator meeting, investigators from each EP demonstrated their daily work at the EP and we recognized that all of the EPs mainly discussed the applicability to genomically matched treatment, including IND trials, and the necessity of genetic counseling. In addition, for all investigators, it was suggested that all pre-reviewing would be carefully carried out by the key members of each EP to make the conference more interactive and to also save time.

### The performance of EPs at each core hospital

A total of 1522 cases (core hospitals, *n* = 747; cooperative hospitals, *n* = 775) underwent CGP tests from June 2019 to January 2020. Among the 747 cases in core hospitals, 28 cases (3.7%, range 0–7.6%) received genomically matched treatments (Table 2); 16 of the 28 cases (2.1%) participated in the IND trials and three cases (0.4%) received genomically-matched treatment as an off-label treatment. The remaining eight cases (1.0%) received approved molecular-targeted drugs. The most common treatments were HER2 inhibitors for *ERBB2* alterations (*n* = 6), followed by FGFR inhibitors for *FGFR/FGF* alterations (*n* = 4), EGFR inhibitors for *EGFR* mutations (*n* = 2), NTRK inhibitors for *NTRK* fusions and mTOR inhibitors for *PIK3CA* mutations (*n* = 2) (Supplementary Table 1). Regarding the germline findings, 17 of 747 cases (2.3%, range 0–15%) were referred for genetic counselling (Supplementary Table 2).

**Table 2:** The performance of each expert panel

Core hospital	No. of patients underwent the CGP test	No. of patients received “matched” therapies	No. of patients referred to genetic counseling
A	75	3	3
B	60	2	0
C	5	0	0
D	41	0	1
E	160	16	5
F	172	4	2
G	13	1	2
H	13	0	0
I	98	0	0
J	24	0	2
K	86	2	3
Total	747	28 (3.7%)	18 (2.4%)

### Clinical annotations of Case 1 (Table 3)

**Table 3:** Clinical annotation for simulated case 1

Core hospital	Recommended therapy	Considered therapy
A	Dabrafenib + trametinib	LXH254, TP0903, olaparib, talazoparib + avelumab, BAY1895344, TAK-931
B	Dabrafenib + trametinib	–
C	Binimetinib + cetuximab + encorafenib	–
D	Dabrafenib + trametinib	–
E	Binimetinib + cetuximab + encorafenib, Dabrafenib + trametinib, talazoparib + avelumab, BAY1895344	–
F	Dabrafenib + trametinib, TP0903, BAY1895344	Trametinib
G	–	Dabrafenib + trametinib
H	Dabrafenib + trametinib	–
I	Binimetinib + cetuximab + encorafenib, Dabrafenib + trametinib, TP0903	–
J	Dabrafenib + trametinib	–
K	Binimetinib + cetuximab + encorafenib, dabrafenib + trametinib, PARP inhibitor	–

*–* not recommended/considered therapies

Nine of 11 EPs recommended dabrafenib (BRAF inhibitor) and trametinib (MEK inhibitor) combination therapy for a *BRAF* V600E mutation. Triplet therapy with encorafenib (BRAF inhibitor), binimetinib (MEK inhibitor), and cetuximab was recommended by four sites (Sites C, E, I and K) because the triplet/doublet (encorafenib plus cetuximab) regimens showed a survival benefit in pre-treated metastatic colorectal cancer patients with *BRAF* V600E mutation in the BEACON global phase 3 trial [13, 14]. Although the ANCHOR global phase 2 trial with the triplet regimen was ongoing in Japan at that time, the eligibility was restricted to chemotherapy-naïve patients; thus, the other seven sites did not recommend it [15]. TP0903, an AXL kinase inhibitor, was recommended by Sites F and I [16]. For *ATM* truncating mutation, two sites (Sites E and F) recommended BAY-1895344, ATR inhibitor [17], and Site E recommended talazoparib plus avelumab [18]. Regarding other candidate therapies, LXH254 (a pan-RAF inhibitor) [19], TP0903, TAK931 (a CDC7 inhibitor) [20], and olaparib (based on a phase 2 trial for advanced solid tumors) [21], and talazoparib + avelumab were considered by Site A, while trametinib was considered by Site F. Site G concluded that there was no recommendation and dabrafenib and trametinib therapy was considered as a treatment option. All 11 sites annotated *BRCA2* V208G as a germline variant of uncertain significance (VUS). Considering the familial history of breast cancers, two EPs (Sites D and H) recommended genetic counseling (Supplementary Table 3).

### Clinical annotations of Case 2 (Table 4)

**Table 4:** Clinical annotation for simulated case 2

Core hospital	Recommended therapy	Considered therapy
A	–	Everolimus + exemestane
B	–	–
C	Trastuzumab deruxtecan	–
D	Everolimus + exemestane, trastuzumab deruxtecan	–
E	Trastuzumab deruxtecan	–
F	–	Everolimus + exemestane
G	–	–
H	Alpelisib	Afatinib
I	Alpelisib, niratinib	–
J	–	Everolimus + exemestane
K	PI3K inhibitor	–

*–* not recommended/considered therapies

One site (Site D) recommended everolimus plus exemestane for *PIK3CA* mutation. That regimen was also considered as a treatment option by Sites A, F and J. Alpelisib or PI3K inhibitor, which are approved by the FDA for hormone receptor-positive breast cancer with *PIK3CA* mutation [22], were recommended by three sites (Sites H, I, and K), but not recommended by the other 8 sites due to closure of the clinical trial in Japan. Trastuzumab deruxtecan was recommended by two sites (Sites C and D) for HER2 IHC1+ [23]. No sites evaluated *ERBB2* S310Y and *CCND1* amplification as druggable. Two sites (Sites B and G) concluded that no genomically matched treatment was available for this patient (Supplementary Table 4).

## Discussion

We reported the initial assessment of the performance of each EP at core hospitals. In the first 8 months after the implementation of reimbursement for CGP tests, 747 cases (ranging 5–172 cases) were tested at 11 core hospitals and 3.7% of these cases received genomically matched treatment. In terms of germline findings, 2.4% of tested cases were referred for genetic counseling. While more than half of these cases participated in IND trials, 11% of cases received matched drugs as an off-label treatment. The findings serve as reference data for assessing the improvement in precision oncology in Japan in the future. We also demonstrate that the clinical annotations of CGP tests varied across EPs, which seemed to be associated with the recognition of the latest information regarding the recruitment status and the eligibility of candidate for IND trials. Because such information cannot be tacked on by the C-CAT, it might be necessary to establish a framework to share the latest information of IND trials across all EPs. In the United States, a virtual molecular tumor board (VMTB), cloud-based computing technology for integrating the CGP results of each patient with a genomic knowledge base to provide an annotation report on candidate trials, showed clinical utility not only for standardizing clinical annotations but also for improving the efficacy of on-site molecular tumor boards [24]. A VMTB may also be an option for Japan.

In previous reports of research-based genomic screening projects, 3–20% of patients received genomically matched treatment on the basis of their CGP test results [25–34]. Our study showed that 3.7% of patients received genomically matched treatment; this percentage seems to be lower in comparison to previous reports. One potential reason for this finding is due to the fact that the CGP tests were mainly reimbursed for patients who had no option other than to receive the best supportive care. Based on the data obtained from both NCI-MATCH and SCRUM-Japan GI SCREEN, nationwide genomic screening projects for refractory patients who have no systemic treatment options, the proportion of patients who enrolled in the matched clinical trials was 2.4% and 2.2% [34, 35], which was similar to the proportion reported in our study. Fundamentally, in addition to refractory patients, precision oncology should also be applied to non-refractory patients. Recently, the “Consensus clinical practice guidance for CGP tests” was updated and now recommends CGP test would be recommended, regardless of the line of treatment [36, 37].

Our study has demonstrated the actual data related to the clinical utility of the CGP test in core hospitals in the clinical setting. However, one limitation associated with our study is due to the fact that the data from cooperative hospitals could not be evaluated since the information regarding the post-expert panel clinical course of cases in these hospitals was not available.

Along with the current increase in the number of CGP tests across Japan, in September 2019, the MHLW established a new designated hospital category, called hub hospitals, which are positioned between core and cooperative hospitals [38]. Hub hospitals are required to establish an EP. Thus, the total number of designated hospitals has recently been expanded to 12 core, 33 hub and 165 cooperative hospitals [38]. Considering the increased numbers of hospitals (core and hub) carrying out EPs, the standardization of clinical annotations has become more relevant to maintaining quality control/assurance of precision oncology in Japan.

We are conducting a further study to develop consensus clinical annotations using another 100 simulated cases covering genomic alterations that are observed with high frequency (approximately 70% of clinical cases) with reference to The Cancer Genome Atlas (TCGA) database (accessed 2 April 2020) [39], as an official educational material for EPs across Japan, and hopefully worldwide. We will also assess the learning effect using this educational material. We believe that our further study will help with the standardization of all EPs.

In conclusion, this is an initial report on the performance of EPs in core hospitals after the implementation of reimbursement for CGP. This report is also the first to raise issues regarding the uniformity of the process and the quality of recommendations that have a great impact on decision-making by patients and attending physicians on EPs at core hospitals. Further investigation on the standardization of EPs is warranted.

## Supplementary Information

Below is the link to the electronic supplementary material.

Supplementary file1 (XLS 70 KB)

## References

[1] National Comprehensive Cancer Network Guidelines (2020). https://www.nccn.org/professionals/physician_gls/default.aspx. Accessed Aug 2020

[2] El-Deiry WS, Goldberg RM, Lenz HJ (2019). The current state of molecular testing in the treatment of patients with solid tumors. CA Cancer J Clin.

[3] Yoshino T, Arnold D, Taniguchi H (2018). Pan-Asian adapted ESMO consensus guidelines for the management of patients with metastatic colorectal cancer: a JSMO-ESMO initiative endorsed by CSCO, KACO, MOS, SSO and TOS. Ann Oncol.

[4] The OncoGuide™ NCC oncopanel system receives insurance coverage for use in cancer genome profiling. https://www.sysmex.co.jp/en/news/2019/190531.html. Accessed Aug 2020

[5] Chugai Launches Genomic Mutation Analysis Program, FoundationOne CDx Cancer Genomic Profile. https://www.chugai-pharm.co.jp/english/news/detail/20190603150001_625.html. Accessed Aug 2020

[6] Ebi H, Bando H (2019). Precision oncology and the universal health coverage system in Japan. JCO Precis Oncol.

[7] https://www.mhlw.go.jp/content/12400000/000514782.pdf. Accessed Aug 2020

[8] https://www.mhlw.go.jp/file/05-Shingikai-10901000-Kenkoukyoku-Soumuka/0000203236.pdf. Accessed Aug 2020

[9] Sunami K (2019). The role of core hospitals and cooperative hospitals for cancer genomic medicine in Japan. Gan To Kagaku Ryoho.

[10] Malone ER, Oliva M, Sabatini PJB (2020). Molecular profiling for precision cancer therapies. Genome Med.

[11] National Cancer Center Center for Cancer Genomics and Advanced Therapeutics. https://www.ncc.go.jp/en/c_cat/about/index.html. Accessed Aug 2020

[12] Sunami K, Takahashi H, Tsuchihara K (2018). Clinical practice guidance for next-generation sequencing in cancer diagnosis and treatment (edition 1.0). Cancer Sci.

[13] Corcoran RB, Atreya CE, Falchook GS (2015). Combined BRAF and MEK inhibition with dabrafenib and trametinib in BRAF V600-mutant colorectal cancer. J Clin Oncol.

[14] Kopetz S, Grothey A, Yaeger R (2019). Encorafenib, binimetinib, and cetuximab in BRAF V600E-mutated colorectal cancer. N Engl J Med.

[15] Grothey A, Tabernero J, Taieb J (2020). ANCHOR CRC: a single-arm, phase 2 study of encorafenib, binimetinib plus cetuximab in previously untreated BRAF V600-mutant metastatic colorectal cancer. Ann Oncol Suppl.

[16] First-in-human study of oral TP-0903 (a novel inhibitor of axl kinase) in patients with advanced solid tumors. https://clinicaltrials.gov/ct2/show/NCT02729298. Accessed Aug 2020

[17] First-in-human study of ATR inhibitor BAY1895344 in patients with advanced solid tumors and lymphomas. https://clinicaltrials.gov/ct2/show/NCT03188965. Accessed Aug 2020

[18] Javelin BRCA/ATM: avelumab plus talazoparib in patients with BRCA or ATM mutant solid tumors. https://clinicaltrials.gov/ct2/show/NCT03565991. Accessed Aug 2020

[19] Phase I study of LXH254 in patients with advanced solid tumors haboring MAPK pathway alterations. https://clinicaltrials.gov/ct2/show/NCT02607813. Accessed Aug 2020

[20] A study to evaluate TAK-931 in participants with advanced nonhematologic tumors. https://clinicaltrials.gov/ct2/show/NCT02699749. Accessed Aug 2020

[21] Efficacy and safety of olaparib (MK-7339) in participants with previously treated, homologous recombination repair mutation (HRRm) or homologous recombination deficiency (HRD) positive advanced cancer (MK-7339-002 / LYNK-002). https://clinicaltrials.gov/ct2/show/NCT03742895. Accessed Aug 2020

[22] André F, Ciruelos E, Rubovszky G (2019). Alpelisib for PIK3CA-mutated, hormone receptor-positive advanced breast cancer. N Engl J Med.

[23] Trastuzumab deruxtecan (DS-8201a) versus investigator’s choice for HER2-low breast cancer that has spread or cannot be surgically removed [DESTINY-Breast04]. https://clinicaltrials.gov/ct2/show/NCT0373402921. Accessed Aug 2020

[24] Pishvaian MJ, Blais EM, Bender RJ (2019). A virtual molecular tumor board to improve efficiency and scalability of delivering precision oncology to physicians and their patients. JAMIA Open.

[25] Naito Y, Takahashi H, Shitara K (2018). Feasibility study of cancer genome alterations identified by next generation sequencing: ABC study. Jpn J Clin Oncol.

[26] Sunami K, Ichikawa H, Kubo T (2019). Feasibility and utility of a panel testing for 114 cancer-associated genes in a clinical setting: a hospital-based study. Cancer Sci.

[27] Tanabe Y, Ichikawa H, Kohno T (2016). Comprehensive screening of target molecules by next-generation sequencing in patients with malignant solid tumors: guiding entry into phase I clinical trials. Mol Cancer.

[28] Kou T, Kanai M, Yamamoto Y (2017). Clinical sequencing using a next-generation sequencing-based multiplex gene assay in patients with advanced solid tumors. Cancer Sci.

[29] Sohal DP, Rini BI, Khorana AA (2015). Prospective clinical study of precision oncology in solid tumors. J Natl Cancer Inst.

[30] Khoury JD, Wang WL, Prieto VG (2018). Validation of immunohistochemical assays for integral biomarkers in the NCI-MATCH EAY131 clinical trial. Clin Cancer Res.

[31] NCI-MATCH precision medicine cancer trial. https://ecog-acrin.org/nci-match-eay131. Accessed Aug 2020

[32] Meric-Bernstam F, Brusco L, Shaw K (2015). Feasibility of large-scale genomic testing to facilitate enrollment onto genomically matched clinical trials. J Clin Oncol.

[33] Trédan O, Wang Q, Pissaloux D (2019). Molecular screening program to select molecular-based recommended therapies for metastatic cancer patients: analysis from the ProfiLER trial. Ann Oncol.

[34] Flaherty KT, Gray R, Chen A (2020). The molecular analysis for therapy choice (NCI-MATCH) trial: lessons for genomic trial design. J Natl Cancer Inst.

[35] Nakamura Y, Taniguchi H, Bando H et al (2020) Clinical utility of circulating tumor DNA sequencing in advanced gastrointestinal cancer: SCRUM-Japan GI-SCREEN and GOZILA studies. Nat Med (in press) 10.1038/s41591-020-1063-5 33020649

[36] Consensus clinical practice guidance for the CGP tests” issued by three cancer-related major Japanese societies (the Japanese Society of Medical Oncology, the Japanese Society of Clinical Oncology, and the Japanese Cancer Association) version 2.0. https://www.jsmo.or.jp/about/kanko.html or http://www.jca.gr.jp/researcher/topics/2020/200310.html. Accessed Aug 2020

[37] Yoshino T, Pentheroudakis G, Mishima S (2020). JSCO-ESMO-ASCO-JSMO-TOS: international expert consensus recommendations for tumour-agnostic treatments in patients with solid tumours with microsatellite instability or NTRK fusions. Ann Oncol.

[38] https://www.mhlw.go.jp/stf/seisakunitsuite/bunya/kenkou_iryou/kenkou/gan/gan_byoin.html. Accessed Aug 2020

[39] cBioPortal for cancer genomics. https://www.cbioportal.org/. Accessed Apr 2020

